# Changes in Circulating Metabolites during Weight Loss and Weight Loss Maintenance in Relation to Cardiometabolic Risk

**DOI:** 10.3390/nu13124289

**Published:** 2021-11-27

**Authors:** Christopher Papandreou, Joanne A. Harrold, Thea T. Hansen, Jason C. G. Halford, Anders Sjödin, Mònica Bulló

**Affiliations:** 1 Institute of Health Pere Virgili, IISPV, University Hospital Sant Joan, 43204 Reus, Spain; christoforos.papandreou@iispv.cat; 2 Department of Biochemistry and Biotechnology, Faculty of Medicine and Health Sciences, Rovira i Virgili University, 43201 Reus, Spain; 3 Department of Psychology, Institute of Population Health, University of Liverpool, Liverpool L69 3GL, UK; harrold@liverpool.ac.uk; 4 Department of Nutrition, Exercise and Sports, Section for Obesity Research, University of Copenhagen, 2200 Copenhagen, Denmark; tha@nexs.ku.dk (T.T.H.); amsj@nexs.ku.dk (A.S.); 5 School of Psychology, University of Leeds, Leeds LS2 9JT, UK; j.halford@leeds.ac.uk; 6 CIBER Fisiopatología de la Obesidad y Nutrición (CIBEROBN), Instituto de Salud Carlos III, 28029 Madrid, Spain

**Keywords:** metabolomics, cardiometabolic risk, weight loss, weight maintenance, SATIN

## Abstract

(1) Background: There is a substantial lack of knowledge of the biochemical mechanisms by which weight loss and weight regain exert their beneficial and adverse effects, respectively, on cardiometabolic outcomes. We examined associations between changes in circulating metabolites and changes in cardiometabolic risk factors during diet-induced weight loss and weight loss maintenance. (2) Methods: This prospective analysis of data from the Satiety Innovation (SATIN) study involved adults living with overweight and obesity (mean age=47.5). One hundred sixty-two subjects achieving ≥8% weight loss during an initial 8-week low-calorie diet (LCD) were included in a 12-week weight loss maintenance period. Circulating metabolites (m=123) were profiled using a targeted multiplatform approach. Data were analyzed using multivariate linear regression models. (3) Results: Decreases in the concentrations of several phosphatidylcholines (PCs), sphingomyelins (SMs), and valine were consistently associated with decreases in total (TChol) and low-density lipoprotein cholesterol (LDL-C) levels during the LCD. Increases in PCs and SMs were significantly associated with increases in TChol and LDL-C during the weight loss maintenance period. Decreases and increases in PCs during LCD and maintenance period, respectively, were associated with decreases in the levels of triglycerides. (4) Conclusions: The results of this study suggest that decreases in circulating PCs and SMs during weight loss and the subsequent weight loss maintenance period may decrease the cardiovascular risk through impacting TChol and LDL-C.

## 1. Introduction

Overweight and obesity represent major health problems worldwide and are associated with increased prevalence of several other chronic conditions, including cardiovascular diseases [1]. Treatment of overweight and obesity is therefore a public health imperative. Even a modest amount of weight loss (5–10%) is known to yield immediate improvements in many cardiometabolic risk factors such as insulin resistance, dyslipidemia, and inflammation [2]. For most, however, maintaining lost weight following structured and intentional weight loss remains a challenge [3,4]. Weight loss maintenance is beneficial for sustaining a favorable cardiometabolic risk profile [5,6], and weight regain is associated with deterioration of cardiometabolic benefits associated with weight loss [6]. However, the underlying mechanisms responsible for the beneficial and adverse effects of weight loss and weight regain, respectively, on cardiometabolic outcomes are not clear yet.

The characterization of the metabolic alterations that are associated with weight changes can provide insights into the mechanisms that lead to overweight/obesity-related comorbidities. Metabolomics, through a systematic evaluation of small molecules in biospecimens such as blood, may help to identify at least part of these alterations. A previous study identified changes in several serum amino acids associated with improvements in insulin resistance in 91 adults living with obesity after one year of a low-calorie diet (LCD) [7]. A more recent study nested within the POUNDS lost trial demonstrated that reductions in plasma choline and trimethylamine N-oxide were associated with improvements in insulin sensitivity after 6months of LCD [8].

Nonetheless, to the best of our knowledge, no study has examined how changes in metabolites during weight loss and weight loss maintenance are related to changes in cardiometabolic risk factors over time. This approach may advance our understanding of potential metabolic pathways underlying the association between weight changes and cardiometabolic health.

Recently, our research group identified associations between changes in circulating lipid species with changes in body weight in participants from the Satiety Innovation (SATIN) study following an eight-week LCD [9]. We also observed improvements in several cardiometabolic risk factors during this period. However, whether changes in metabolites’ concentrations were also associated with improvements in participants’ cardiometabolic risk profile during weight loss and weight regain, respectively, has not been examined. Therefore, we investigated associations between changes in circulating metabolites and changes in metabolic traits, lipid profile, and inflammation during diet-induced weight loss and weight loss maintenance in the SATIN study.

## 2. Subjects and Methods

### 2.1. Study Design and Participants

The current analysis nested within the FP-7 European Commission project SATIN work package 5 (NCT02485743), a randomized multicenter (Denmark, Spain) trial designed to examine the effect of enhancing satiety on weight regain prevention.The design and methods of SATIN have been detailed in [9,10,11]. Briefly, women and men aged between 20 and 65 years with an initial body mass index (BMI) of 27.0 to 35.0 kg/m^2^, fat mass of no less than 23%, and without comorbidities were recruited and were instructed to follow the Modifast^®^ (Nutrition et Santé, Revel, France) formula diet to achieve at least an 8% of weight reduction over an initial 8-week low-calorie diet (LCD) period. Participants reaching the pre-defined weight loss were randomly allocated to one of the two intervention groups for 12weeks (weight loss maintenance period): (1) including an active satiety-enhancing product (active intervention group) or (2) including a similar control product without satiety-enhancing properties (control group) [10,11].

The protocol of the SATIN trial was in accordance with the Declaration of Helsinki (Fortaleza, Brazil, October 2013), and it was approved by the local institutional review boards and Ethics Committees in the two recruiting centers (15-07-30/7assN2 for the Spanish centre and H-15008553 for the Danish centre). All participants provided written informed consent.

Of the 236 participants recruited at baseline and with available blood samples, a total of 162 participants achieved >8% weight loss and randomized to the weight loss maintenance period (75 subjects from Reus and 87 from Copenhagen, Figure S1). Seven participants were further excluded because of lack of adiposity measures after the weight loss maintenance period (Figure S1).

### 2.2. Assessment of Energy Intake and Physical Activity

Nutritional data were collected at baseline and at the end of each period using 3-day dietary records. Energy intakes were calculated using Danish and Spanish food composition tables [12].

Total physical activity was assessed with an ActiGraph™ tri-axis accelerometer monitor (GT3X+) over seven consecutive days at baseline and at the end of the 8 weeks of LCD and the 12-week weight loss maintenance period, respectively. Data were expressed as vector magnitude of the total tri-axial counts from monitor wear time divided by monitor wear time.

### 2.3. Anthropometric and Biochemical Measurements

Anthropometric measures including body weight, height, and sagittal abdominal diameter were determined by trained personnel at baseline, after the 8 weeks of LCD, and at the end of a 12-week weight loss maintenance period. BMI was calculated. Blood samples were collected in fasting conditions at the three time points. Glucose and insulin concentrations were measured using standard enzymatic automated methods, and the insulin resistance index (HOMA-IR) was estimated [13]. Total cholesterol (TChol), high-density lipoprotein (HDL-C) cholesterol, low-density lipoprotein (LDL-C) cholesterol, and triglycerides levels were measured using standard enzymatic automated methods (COBAS; Roche Diagnostics Ltd., Rotkreuz, Switzerland). Inflammatory markers including interleukin 6 (IL-6) and C-reactive protein (CRP) were assessed using MagPix platform (Luminex Corporation, Austin, TX, USA).

### 2.4. Multiplatform Targeted Metabolomics

Metabolites were analysed at baseline, after each period, using a multiplatform approach including gas chromatography coupled to high resolution mass spectrometry (GC-HRMS), liquid chromatography coupled to high resolution mass spectrometry (LC-HRMS), and proton nuclear magnetic resonance (^1^H-NMR). The analytical procedures are specified in the Supplemental Methods. Information about the mass to charge ratio, retention time, repeatability, and reproducibility (expressed as RSD) for each metabolite is shown in Table S1.

### 2.5. Statistical Analyses

Baseline results were expressed as mean ± standard deviation (SD) for continuous variables and percentages for categorical variables. Changes in baseline characteristics after the 8-week and 12-week periods are expressed as mean (95% confidence interval (CI)), and their statistical significance was evaluated using a paired *t*-test. Individual metabolites with equal or more than 20% missing values were excluded [14], leaving 123 metabolites for further analyses. Data on metabolites were log-transformed to improve normality. We first analysed changes in metabolites concentrations between baseline and the 8-week LCD period using a paired *t*-test. Two-sided *p* values were reported according to an alpha level = 0.0004 (alpha = 0.05 with Bonferroni correction for 123 independent tests (including 123 metabolites). For those metabolites found to change significantly, we assessed their associations with improvements in cardiometabolic parameters (glucose, HOMA-IR, TChol, HDL-C, LDL-C, triglycerides, CRP, and IL-6) over the 8-week LCD period. With respect to metabolites, we first calculated the difference between 8-week log-transformed value and baseline log-transformed value and then scaled these differences to multiples of 1 SD. Linear regression models were fitted to examine these associations adjusting for age, sex, body weight change, sagittal diameter change, value for the respective outcome traits at the baseline examination, and the respective metabolite at baseline. Since IL-6 levels did not significantly change over this period (see Table 1), we did not examine its association with metabolites. The Bonferroni correction for 60 (in case of CRP), 59 (in case of glucose, HOMA-IR), 58 (in case of triglycerides), and 57 (in case of TChol, LDL-C, and HDL-C) independent tests for each respective outcome trait was applied, and significance was reported according to an alpha level = 0.0008 (alpha corrected according to the significant changes in Table 2). To test the robustness of these associations, we conducted a sensitivity analysis, further adjusting the multivariable model for changes in energy intake and physical activity over 8 weeks. Metabolites found to be consistently associated with TChol and LDL-C were further investigated by metabolite set enrichment analysis using MetaboAnalyst 5.0. A metabolite set enrichment analysis was also used for those metabolites significantly associated with triglycerides. One-tailored *p*-values were provided after adjusting for multiple testing (false discovery rate method). Thereafter, we analysed changes in those metabolites, found to associate significantly with improvements in cardiometabolic parameters, between the end of 8 weeks and the 12-week weight loss maintenance period using a paired *t*-test. Two-sided *p* values were reported according to an alpha level = 0.001 (alpha = 0.05 with Bonferroni correction for 28 independent tests (including 28 metabolites)). To test whether changes in these metabolites were significantly associated with changes in cardiometabolic parameters (TChol, HDL-C, LDL-C, and triglycerides) that significantly changed over the weight loss maintenance period, linear regression models were fitted for each outcome trait. Multivariate-adjusted models were performed, including age, sex, body weight change, sagittal diameter change, change in the respective outcome traits over 8 weeks, intervention group (enhancing satiety foods and control foods), and change in the respective metabolite over 8 weeks. Bonferroni correction for the number of tests based on the number of metabolites found to associate significantly with cardiometabolic measures was applied (alpha corrected for TChol, LDL-C, and triglycerides in the main Table 3). Statistical analyses were performed using Stata 14.1 ((Stata Corporation, College Station, TX, USA)).

## 3. Results

### 3.1. Characteristics of the Study Participants

Anthropometric, biochemical, nutritional, and physical activity data are shown in Table 1. The study population had a mean age of 47.3 ± 9.9 years. Their body weight and BMI were 88.1 ± 10.7 kg and 30.9 ± 2.0 kg/m2, respectively, at baseline. No significant differences in the general characteristics of the 162 participants included in the present analyses and those participants initially recruited in the SATIN study were observed (Table S2). The average weight loss and reduction in sagittal abdominal diameter for the 162 participants achieving ≥8% during the LCD were 9.7 kg and 3.1 cm, respectively (*p* <0.001). Regarding metabolic parameters, glucose levels, insulin levels, and HOMA-IR decreased by 2.9 mg/dL, 3.2 mcUI/mL, and 0.8 units, respectively. TChol, HDL-C, LDL-C, triglycerides, and CRP also decreased by 19.0, 5.2, 12.3, 11.6, and 0.34 mg/L, respectively. Furthermore, total energy intake and physical activity significantly decreased and increased, respectively. IL-6 did not significantly change.

After the 12-week weight loss maintenance period, 155 participants with available adiposity measures regained, on average, 1.0 kg (*p*<0.001). The sagittal diameter also increased (0.6 cm) (*p*<0.001). During this period, TChol, HDL-C, and LDL-C increased by 15.1, 10.3, and 5.9 mg/dL, whereas triglycerides decreased by 6.5 mg/dL.

Other metabolic and inflammatory markers, as well as energy intake and physical activity, did not significantly change.

### 3.2. Association between Changes in Metabolite Concentrations and Changes in Cardiometabolic Parameters over the 8-Week LCD

After correction for multiple testing, 60 metabolites showed a significant change from baseline to the end of the 8-week LCD period (Table S3). Changes in metabolite concentrations associated with changes in adiposity measures are shown in Table 2.

Weight-loss-induced decreases in triglycerides (TG), phosphatidylcholines (PCs), lysophosphatidylcholines (LPCs), sphingomyelins (SMs), fatty acid chains (FAC), and monounsaturated fatty acids (MUFA) (Table S3) were associated with decreased levels of TChol and LDL-C. Decreases in lipid species including PCs (33:1, 35:1, 36:1, 36:4e, 38:3, 38:4, 40:6), SMs (32:1, 32:2, 33:1, 35:1, 36:1, 38:1, 40:1, 40:2, 41:1, 41:2, 42:1), and TG 50:2 (Table S3) were associated with decreases in TChol and LDL-C. On the contrary, increases in SM 42:3 were associated with decreases in LDL-C. Additionally, decreases in LPC and SM 40:2 were associated with decreases in HDL-C, whereas increases in PC 34:2e were associated with decreases in both HDL-C and HOMA-IR. Associations between changes in seven PCs (32:1, 33:1, 35:1, 36:1, 38:3, 40:6, 34:2e), and triglycerides were also found.

**Figure 1:**
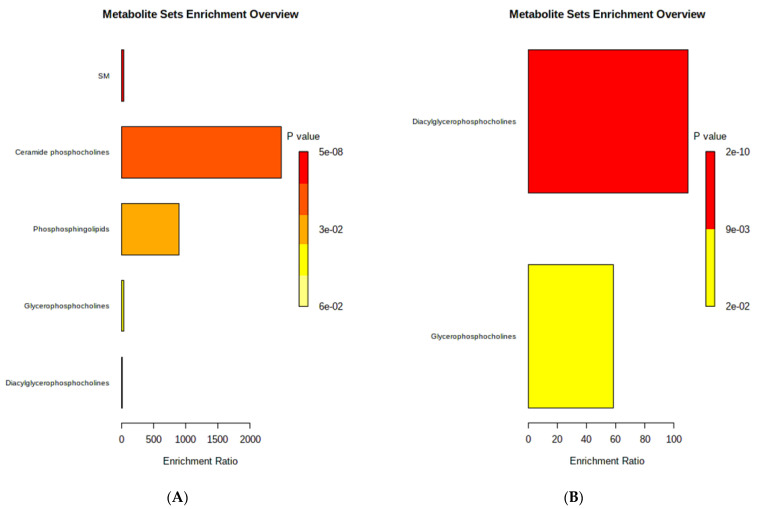
Metabolite set enrichment analysis. (**A**) Over representation analysis was implemented in the enrichment analysis of MetaboAnalyst 5.0 using the hypergeometric test to evaluate whether a particular metabolite set is represented more than expected by chance within the metabolites previously selected for TChol and LDL-C. One-tailed *p* values are provided after false discovery rate-adjusting for multiple testing. Abbreviations: SM, sphingomyelin. (**B**) Over-representation analysis was implemented in the enrichment analysis of MetaboAnalyst 5.0 using the hypergeometric test to evaluate whether a particular metabolite set is represented more than expected by chance within the metabolites previously selected for triglyceride. One-tailed *p* values are provided after false discovery rate-adjusting for multiple testing.

The top enriched metabolites for TChol and LDL-C were selected by a threshold of false discovery rate-adjusted *p* value < 0.05 and included SMs, ceramide PCs, phosphosphingolipids, glycerophosphocholines, and diacylglycerophosphocholines (Figure 1A). Diacylglycerophosphocholines and glycerophosphocholines were selected as the top enriched metabolites for triglycerides (Figure 1B). In sensitivity analyses, these associations persisted after adjustment for changes in total energy intake and physical activity (Table S4). Furthermore, the associations between decreases in the concentrations of valine and TChol, and LDL-C were significant after these adjustments (Table S4). On the other hand, the association between PC 34:2e and HOMA-IR was attenuated. No significant associations between the measured metabolites and glucose or CRP were observed.

### 3.3. Association between Changes in Metabolite Concentrations and Changes in Cardiometabolic Parameters over the 12-Week Weight Loss Maintenance Period

After correction for multiple testing, 22 lipid species showed a significant change over the 12-week weight loss (Table S5). We found increases in the concentrations of several PCs (33:1, 36:1, 36:4e, and 38:3) and SMs (32:1, 32:2, 33:1, 38:1, 40:1, 40:2, 41:1, 41:2, and 42:1) associated with increases in the levels of TChol over the maintenance period (Table 3). A similar trend in the associations of PC 36:4e, PC 38:3, and SMs including SM 32:1, SM 32:2, SM 33:1, SM 38:1, SM 40:1, SM 40:2, SM 41:1, SM 42:1, and SM 42:3 with increases in LDL-C were also observed. Furthermore, PC 32:1, PC 33:1, and PC 36:1 were found to be associated with decreases in triglycerides levels. There were no significant associations between metabolites and HDL-C.

## 4. Conclusions

In the present study, we found that decreases in several circulating PCs and SMs, over an 8-week LCD, were consistently and independently associated with reductions in TChol and LDL-C serum levels. Our study also demonstrated for the first time that increases in these lipid species over a 12-week period of weight loss maintenance, in which on average subjects experienced a small but significant weight regain, were related to a deterioration of this lipid profile. Our results also indicated that changes in PCs over both periods were associated with decreases in the serum levels of triglycerides.

A cycle of weight regain following intentional weight loss has been associated with increased cardiovascular risk [15]. In a previous prospective study, after a weight loss period of 5 months and associated reduction in the levels of TChol, LDL-C, glucose, and HOMA-IR, postmenopausal women who regained at least 2 kg of lost weight within a year experienced an increase of their levels [16]. Similarly, we observed a trend towards reductions in these cardiometabolic traits and CRP after weight loss, while the levels of TChol and LDL-C increased after a mean weight regain of 1 kg (approximately 10%) over the weight loss maintenance period. Whether other factors other than weight change may be driving these changes remains unknown.

Many phospholipids and sphingolipids have been implicated as critical components linking obesity to cardiometabolic diseases. Obesity results in a generally higher lipid load in circulation characterized by increased PC and SM species [17,18], while elevated circulating PC and SM levels have been suggested to increase the risk of coronary artery disease and mortality [19]. Previous weight loss studies found decreases in their levels [9,20], but whether these changes would partially explain the beneficial health effects of weight loss [21] is still unclear. In our study, PC, the most abundant lipid subclass in LDL-C [22], decreased after 8 weeks of an LCD and was associated with decreases in TChol and LDL-C levels. Sphingolipids are a class of lipids found predominantly in circulating LDL-C [22] and activate inflammatory pathways [23]. Higher levels of sphingolipids are associated with obesity and related co-morbidities [24]. We observed a decrease in SMs, the most abundant of the sphingolipids, after the LCD, which is in line with results reported after lifestyle interventions in children [25] and adults [26] living with overweight and obesity. Additionally, several SMs mainly consisted of monounsaturated and polyunsaturated species were associated with decreases in TChol and LDL-C. We suggest that a decrease in the biosynthesis of these SM species could indicate a better LDL-C profile and consequently a potential reduction in the risk of developing atherosclerosis [27]. Interestingly, the associations of PCs and SMs with LDL-C were independent of decreases in body weight and sagittal abdominal diameter; hence, the phosphosphingolipid composition of LDL-C might hold valuable insights into the biological mechanisms behind the effects of the weight loss on cardiovascular risk. On the other hand, increases in these lipid species were related to a deterioration of the lipid profile during weight regain independently of increases in body weight and visceral fat. It has been demonstrated that weight regain exerts adverse effects on cardiovascular health [15], and our results add to the evidence that changes in PCs and SMs may play a role.

Branched-chain amino acids (BCAAs) have been associated with the risk of developing atherosclerosis [28]. Previous studies suggested that circulating BCAAs are associated with CVD predominantly through type 2 diabetes-related pathways [29]. Furthermore, increased BCAA levels have been associated with elevated triglyceride levels and reduced HDL-C levels, which are involved in insulin resistance [30]. Previous findings indicated that weight loss decreases circulating BCAAs [31,32]. Our study found decreases in valine concentrations over an LCD associated with improvements in TChol and LDL-C levels, but not HOMA-IR, triglycerides, and HDL-C. Regarding the relationship between BCAAs and LDL-C, Halama et al. [33] reported that the by-products of leucine degradation could serve as a substrate for cholesterol biosynthesis in vitro. Our results add to the growing body of evidence that BCAA levels could be involved in the regulation of lipid metabolism [34].

In our study, initial decreases in triglycerides over the LCD were sustained during the maintenance period, despite a 1 kg regain, and this is in line with two previous trials despite 2% and 6% weight regain [35,36]. The maintenance of triglyceride improvements in our study versus others [37,38] could be explained by differences in the length of the weight loss maintenance period evaluated. We and the two aforementioned trials implemented a rather shorter maintenance period (12–24 weeks) compared to other trials (52–104 weeks) [37,38], and we could speculate that our short period might not be long enough to allow for triglycerides to increase. We also observed independent associations between decreases in several PCs and triglycerides during weight loss. Experimental studies suggested an essential role of PC biosynthesis in triglyceride secretion from liver [39]. Therefore, we speculate that moderate weight loss induced by an LCD might have improved serum triglyceride levels, possibly through decreases in PC synthesis. However, over the maintenance period, changes in PC concentrations were not associated in the same direction with changes in serum triglycerides, and other factors such as the length of this period might explain this observation.

Strengths of this study include repeated measures and quantification of a wide range of metabolites using combinations of different metabolomic platforms. In addition, our study participants were free of cardiometabolic diseases and were non-smokers, thus avoiding their effect on the concentrations of metabolites. The consistent findings of the PCs and SMs with TChol and LDL-C during the LCD and weight loss maintenance period strengthen our conclusion.

Concerning limitations, we recruited participants without comorbidities, and this might limit the generalizability of our findings to individuals with obesity-associated comorbidities. Furthermore, due to the observational nature of this study, there is the possibility of unmeasured residual confounding. Finally, the use of a targeted metabolomic approach only may have partially covered the blood metabolome, limiting the identification of new metabolites associated with changes in cardiometabolic risk factors. Supplementing the current study with complementary untargeted metabolomics would help provide a more comprehensive view of the metabolic changes that accompany weight loss and weight regain and could be associated with cardiometabolic parameters.

In summary, decreases in circulating concentrations of PCs, SMs, and valine were associated with improvements in TChol and LDL-C in adults living with overweight and obesity following an LCD. Furthermore, decreases in PC concentrations were associated with decreases in triglycerides levels. On the other hand, positive associations between changes in PCs, SMs, TChol, and LDL-C were found after weight regain. These data are intriguing in light of a growing body of evidence suggesting that weight loss is associated with improvements in cardiovascular risk, while weight regain is associated with deterioration. Changes in these lipid species in relation to changes in the lipid profile during weight loss and regain could help us to understand the potential role of metabolic responses in cardiovascular health. Our results also suggest that PCs and SMs could be potential biomarkers for successful reductions in cardiovascular risk factors in diet-induced weight loss interventions and subsequent weight loss maintenance.

## Figures and Tables

**Table 1:** General characteristics of the study subjects at baseline, after the 8-week LCD, and after the 12-week weight loss maintenance period.

	Baseline (n = 162)	8 Weeks Change (n = 162)	Weight Loss Maintenance Change (n = 155)	*p* Value for 8 Weeks Change	*p* Value for Weight Loss Maintenance Change
Sex (%Women)	75.0	NA	NA	NA	NA
Age (years)	47.5 ± 9.9	NA	NA	NA	NA
Height (m)	1.68 ± 0.09	NA	NA	NA	NA
Weight (kg)	88.1 ± 10.7	−9.7 (−10.2, −9.2)	1.0 (0.6, 1.4)	<0.001	<0.001
BMI (kg/m^2^)	30.9 ± 2.0	−3.4 (−3.5, −3.2)	0.3 (0.2, 0.5)	<0.001	<0.001
Sagittal diameter (cm)	23.2 ± 2.4	−3.1 (−3.3, −2.8)	0.6 (0.4, 0.8)	<0.001	<0.001
Glucose (mg/dL)	94.9 ± 11.2	−2.9 (−4.1, −1.8)	−0.5 (−1.5, 0.6)	<0.001	0.367
Insulin (mcUI/mL)	9.1 ± 6.4	−3.2 (−3.9, −2.5)	0.2 (−0.2, 0.7)	<0.001	0.361
HOMA-IR	2.2 ± 1.7	−0.8 (−1.0, −0.6)	0.04 (−0.07, 0.1)	<0.001	0.482
TChol (mg/dL)	197.6 ± 34.9	−19.0 (−23.4, 16.2)	15.1 (11.6, 18.6)	<0.001	<0.001
HDL-C (mg/dL)	56.3 ± 15.9	−5.2 (−6.5, −3.8)	10.3 (8.9, 11.7)	<0.001	<0.001
LDL-C (mg/dL)	120.6 ± 30.9	−12.3 (−15.5, −9.2)	5.9 (2.9, 8.9)	<0.001	<0.001
Triglycerides (mg/dL)	103.1 ± 50.5	−11.6 (−18.0, −5.1)	−6.5 (−11.6, −1.4)	<0.001	0.012
IL-6 (pg/mL)	2.24 ± 3.43	−0.33 (−0.75, 0.21)	−0.04 (−0.32, 0.24)	0.125	0.762
CRP (mg/mL)	1.41 ± 1.61	−0.34 (−0.57, −0.10)	−0.008 (−0.23, 0.21)	0.005	0.941
EI (Kcal/d)	1953.5 ± 633.5	−400.8 (−502.3, −299.2)	57.5 (−26.7, 141.8)	<0.001	0.179
TPA (CPM)	608.9 ± 187.9	47.9 (23.5, 72.4)	−14.9 (−46.4, 16.5)	<0.001	0.348

Baseline data are presented as mean ± standard deviation unless otherwise indicated. Data are presented as mean (95% confidence interval) for changes after 8 weeks of LCD and changes between the end of the 8-week intervention and the conclusion of the 12-week weight maintenance period. The paired *t*-test was used to assess changes in variables between baseline and 8 weeks of LCD and after the 12 weeks of the weight loss maintenance period. Abbreviations: BMI, body mass index; CPM, counts/min; CRP, C-reactive protein; EI, energy intake; HDL-C, high-density lipoprotein-cholesterol; IL-6, interleukin 6; LCD, low-calorie diet; LDL-C, low-density lipoprotein-cholesterol; TChol, total cholesterol; TPA, total physical activity.

**Table 2:** Changes in cardiometabolic parameters at 8 weeks of a low-calorie diet (LCD) per 1SD log-transformed changes in the concentrations of metabolites.

Change in Metabolite between Baseline and 8 Weeks LCD	Change in Glucose (mg/dL)	Change in HOMA-IR	Change in TChol (mg/dL)	Change in LDL-C (mg/dL)	Change in HDL-C (mg/dL)	Change in Triglycerides (mg/dL)	Change in CRP (mg/mL)
Free Chol	−0.08 (−1.16, 1.01)	0.04 (−0.09, 0.17)	NA	NA	NA	7.93 (2.62, 13.24)	−0.18 (−0.38, 0.03)
Esterified Chol	0.01 (−1.01, 1.03)	0.06 (−0.06, 0.18)	NA	NA	NA	6.46 (1.44, 11.49)	−0.21 (−0.40, −0.01)
Total Chol	−0.93 (−1.90, 0.05)	−0.05 (−0.17, 0.08)	NA	NA	NA	−1.51 (−6.55, 3.54)	0.07 (−0.12, 0.26)
TG	−0.04 (−1.14, 1.05)	0.18 (0.04, 0.31)	11.16 (7.65, 14.67) **	6.90 (3.60, 10.20) **	−0.78 (−2.04, 0.49)	NA	0.05 (−0.16, 0.26)
PC	−1.10 (−1.16, 0.95)	0.05 (−0.07, 0.19)	13.55 (10.45, 16.65) **	9.85 (6.92, 12.77) **	1.82 (0.60, 3.04)	9.22 (4.09, 14.34)	−0.19 (−0.39, 0.007)
LPC	−0.07 (−1.12, 0.98)	0.08 (−0.05, 0.21)	13.60 (10.57, 16.63) **	9.11 (6.17, 12.06) **	2.32 (1.13, 3.51) **	10.46 (5.44, 15.49) ***	−0.22 (−0.42, −0.02)
SM	−0.39 (−1.49, 0.72)	−0.03 (−0.17, 0.10)	7.93 (4.04, 11.83) **	7.49 (4.14, 10.83) **	0.34 (−0.98, 1.66)	1.18 (−4.46, 6.82)	−0.10 (−0.31, 0.11)
FAC	0.003 (−1.17, 1.18)	0.11 (−0.02, 0.26)	14.18 (10.69, 17.67) **	9.82 (6.52, 13.11) **	0.77 (−0.59, 2.13)	17.45 (12.31, 22.59) ***	−0.01 (−0.23, 0.21)
MUFA	−0.35 (−1.26, 0,56)	−0.005 (−0.12, 0.11)	8.63 (5.66, 11.59) *	7.90 (5.32, 10.49) **	0.52 (−0.58, 1.64)	3.75 (−0.87, 8.38)	−0.12 (−0.30, 0.05)
LPC 14:0	1.17 (−0.13, 2.47)	0.25 (0.09, 0.41)	5.96 (1.30, 10.62)	2.95 (−1.21, 7.10)	0.85 (−0.74, 2.44)	8.11 (1.43, 14.78)	−0.14 (−0.40, 0.11)
LPC 20:3	−0.23 (−1.39, 0.93)	0.13 (−0.01, 0.27)	5.80 (1.81, 9.78)	3.61 (0.03, 7.19)	0.68 (−0.70, 2.07)	6.96 (1.17, 12.74)	−0.10 (−0.33, 0.12)
PC 30:0	1.25 (−0.08, 2.58)	0.25 (0.09, 0.42)	7.58 (2.91, 12.25)	3.94 (−0.30, 8.17)	1.00 (−0.64, 2.64)	9.84 (3.02, 16.67)	−0.09 (−0.36, 0.17)
PC 32:1	0.23 (−0.92, 1.38)	0.18 (0.04, 0.32)	6.39 (2.43, 10.34)	2.18 (−1.44, 5.80)	1.03 (−0.37, 2.44)	12.89 (7.17, 18.60) ***	−0.06 (−0.28, 0.17)
PC 32:2	1.42 (0.18, 2.67)	0.20 (0.04, 0.35)	7.46 (3.07, 11.85)	3.47 (−0.50, 7.44)	1.72 (0.23, 3.21)	10.39 (4.16, 16.61)	−0.23 (−0.47, 0.01)
PC 33:1	0.48 (−0.75, 1.71)	0.14 (−0.01, 0.29)	8.44 (4.24, 12.64) **	5.05 (1.22, 8.88)	0.20 (−1.32, 1.72)	13.78 (7.81, 19.74) ***	−0.16 (−0.40, 0.08)
PC 34:4	1.71 (0.40, 3.01)	0.25 (0.08, 0.41)	8.26 (3.63, 12.90)	3.97 (−0.22, 8.16)	2.04 (0.47, 3.61)	10.57 (3.96, 17.18)	−0.18 (−0.44, 0.07)
PC 35:1	0.47 (−0.75, 1.69)	0.13 (−0.02, 0.28)	10.44 (6.35, 14.53) **	7.81 (4.16, 11.47) **	−0.23 (−1.69, 1.23)	12.36 (6.46, 18.25) ***	−0.22 (−0.45, 0.01)
PC 36:1	0.18 (−1.05, 1.41)	0.16 (0.01, 0.31)	9.56 (5.51, 13.61) **	5.95 (2.21, 9.70)	1.04 (−0.42, 2.51)	11.28 (5.23, 17.33) ***	−0.08 (−0.32, 0.15)
PC 36:4e	0.50 (−0.82, 1.82)	0.002 (−0.16, 0.16)	8.51 (3.99, 13.02) **	7.66 (3.72, 11.59) **	2.16 (0.62, 3.69)	−6.85 (−13.40, −0.31)	−0.18 (−0.43, 0.07)
PC 36:5	−0.05 (−1.06, 0.96)	0.03 (−0.09, 0.16)	4.72 (1.19, 8.24)	3.05 (−0.08, 6.19)	0.78 (−0.42, 1.99)	3.06 (−2.05, 8.17)	−0.04 (−0.23, 0.15)
PC 38:3	0.73 (−0.35, 1.82)	0.19 (0.06, 0.32)	10.14 (6.67, 13.60) **	6.81 (3.57, 10.05) **	0.71 (−0.59, 2.01)	13.66 (8.54, 18.78) ***	0.06 (−0.15, 0.27)
PC 38:4	−0.35 (−1.58, 0.89)	0.09 (−0.06, 0.23)	7.50 (3.48, 11.53) **	5.92 (2.20, 9.64)	0.17 (−1.19, 1.53)	8.32 (2.29, 14.36)	0.19 (−0.02, 0.40)
PC 38:4e	1.14 (0.01, 2.28)	0.01 (−0.13, 0.16)	7.06 (3.06, 11.07)	6.59 (3.08, 10.09) **	1.84 (0.48, 3.20)	−7.56 (−13.36, −1.77)	−0.20 (−0.42, 0.02)
PC 40:4	−0.04 (−1.25, 1.17)	0.12 (−0.03, 0.27)	7.44 (3.24, 11.63)	3.99 (0.18, 7.80)	1.33 (−0.12, 2.79)	10.16 (4.14, 16.19)	−0.03 (−0.26, 0.21)
PC 40:6	−0.07 (−1.09, 0.94)	0.13 (0.004, 0.26)	8.63 (5.18, 12.09) **	5.42 (2.26, 8.57)	0.83 (−0.41, 2.07)	10.33 (5.31, 15.34) ***	−0.004 (−0.20, 0.19)
PE 36:5e	0.48 (−0.61, 1.57)	0.05 (−0.08, 0.19)	0.08 (−3.82, 3.97)	0.84 (−2.58, 4.26)	0.46 (−0.83, 1.76)	−4.87 (−10.34, 0.60)	−0.08 (−0.29, 0.12)
PE 38:5e	0.42 (−0.71, 1.56)	0.04 (−0.10, 0.19)	2.88 (−1.17, 6.93)	3.20 (−0.36, 6.75)	0.92 (−0.42, 2.27)	−6.06 (−11.75, −0.37)	−0.15 (−0.37, 0.07)
PE 38:6e	0.34 (−0.91, 1.59)	−0.03 (−0.18, 0.13)	3.58 (−0.89, 8.05)	3.15 (−0.78, 7.08)	2.03 (0.56, 3.49)	−6.52 (−12.82, −0.22)	−0.25 (−0.49, −0.01)
SM 32:1	−0.06 (−1.24, 1.11)	0.01 (−0.13, 0.16)	14.51 (10.92, 18.10) **	11.73 (8.50, 14.97) **	2.31 (0.95, 3.67)	3.08 (-2.89, 9.05)	−0.15 (−0.38, 0.07)
SM 32:2	0.20 (−1.10, 1.50)	0.14 (−0.01, 0.30)	11.92 (7.67, 16.17) **	8.48 (4.59, 12.37) **	1.76 (0.27, 3.26)	10.68 (4.27, 17.10)	−0.09 (−0.35, 0.16)
SM 33:1	−0.09 (−1.24, 1.07)	−0.02 (−0.16, 0.12)	12.66 (9.02, 16.30) **	10.98 (7.78, 14.18) **	1.86 (0.52, 3.20)	0.36 (−5.52, 6.24)	−0.20 (−0.42, 0.01)
SM 35:1	−0.10 (−1.17, 0.97)	−0.07 (−0.20, 0.07)	10.46 (6.93, 13.98) **	9.12 (6.04, 12.20) **	1.44 (0.18, 2.71)	−0.29 (−5.76, 5.17)	−0.10 (−0.31, 0.10)
SM 36:0	0.30 (−0.95, 1.56)	−0.03 (−0.18, 0.13)	3.46 (−1.16, 8.08)	4.90 (0.95, 8.85)	−0.50 (−1.99, 1.00)	−1.57 (−7.88, 4.75)	0.06 (−0.17, 0.31)
SM 36:1	−0.41 (−1.50, 0.67)	−0.10 (−0.23, 0.03)	9.06 (5.34, 12.78) **	7.87 (4.66, 11.08) **	1.09 (−0.20, 2.37)	1.10 (−4.43, 6.64)	−0.07 (−0.28, 0.14)
SM 38:1	0.63 (−0.43, 1.70)	0.04 (−0.09, 0.17)	10.64 (7.20, 14.08) **	8.19 (5.08, 11.31) **	1.39 (0.14, 2.65)	6.31 (0.97, 11.65)	−0.13 (−0.33, 0.08)
SM 40:1	0.37 (−0.78, 1.52)	0.03 (−0.11, 0.18)	14.71 (11.21, 18.21) **	11.92 (8.72, 15.11) **	2.09 (0.74, 3.44)	7.40 (1.62, 13.17)	−0.23 (−0.45, −0.01)
SM 40:2	0.24 (−0.92, 1.39)	0.02 (−0.12, 0.17)	14.63 (11.19, 18.07) **	11.45 (8.30, 14.60) ***	2.55 (1.24, 3.87) **	4.53 (−1.32, 10.39)	−0.30 (−0.52, −0.08)
SM 41:1	0.51 (−0.71, 1.74)	0.04 (−0.11, 0.19)	15.29 (11.60, 18.99) **	12.51 (9.15, 15.87) **	1.87 (0.43, 3.31)	7.05 (0.91, 13.18)	−0.23 (−0.47, −0.0008)
SM 41:2	−0.20 (−1.69, 1.29)	−0.03 (−0.21, 0.16)	10.55 (5.45, 15.65) **	8.02 (3.46, 12.57)	1.50 (−0.27, 3.26)	6.95 (−0.54, 14.45)	−0.37 (−0.65, −0.09)
SM 42:1	0.62 (−0.63, 1.87)	0.07 (−0.08, 0.22)	12.48 (8.39, 16.57) **	9.47 (5.72, 13.23) **	1.88 (0.41, 3.35)	9.00 (2.83, 15.18)	−0.19 (−0.43, 0.05)
TG 50:2	0.16 (−0.92, 1.23)	0.14 (0.004, 0.27)	6.92 (3.18, 10.66) **	4.09 (0.71, 7.47)	−0.94 (−2.20, 0.31)	NA	0.06 (−0.15, 0.27)
Lactic acid	0.68 (−0.40, 1.77)	0.11 (−0.03, 0.24)	2.73 (−1.17, 6.62)	1.48 (−1.97, 4.93)	0.63 (−0.68, 1.94)	3.30 (−2.25, 8.86)	0.05 (−0.16, 0.26)
Glycolic acid	0.17 (−1.03, 1.38)	0.11 (−0.04, 0.26)	0.16 (−4.16, 4.50)	0.05 (−3.72, 3.84)	−0.05 (−1.49, 1.39)	−2.90 (−9.13, 3.32)	−0.09 (−0.32, 0.13)
Valine	0.50 (−0.58, 1.58)	0.11(−0.02, 0.24)	6.45 (2.73, 10.16)	5.57 (2.31, 8.83)	−0.63 (−1.93, 0.66)	6.38 (0.92, 11.83)	−0.06 (−0.27, 0.15)
Glutamate	0.99 (−0.03, 2.01)	0.12 (−0.01, 0.24)	−0.32 (−4.01, 3.37)	−0.61 (−3.85, 2.63)	−0.38 (−1.62, 0.85)	3.11 (−2.03, 8.26)	0.002 (−0.20, 0.20)
Glucose	NA	NA	1.09 (−2.87, 5.06)	1.47 (−2.02, 4.97)	0.37 (−0.96, 1.70)	−2.64 (−8.30, 3.02)	−0.02 (−0.24, 0.19)
Tyrosine	1.13 (0.07, 2.19)	0.18 (0.05, 0.32)	3.22 (−0.56, 7.01)	2.09 (−1.26, 5.45)	0.63 (−0.65, 1.91)	2.47 (−2.99, 7.92)	−0.13 (−0.34, 0.07)
LPC 16:0	−1.03 (−2.27, 0.21)	0.03 (−0.13, 0.19)	6.52 (2.12, 10.92)	4.77 (0.85, 8.69)	0.54 (−0.95, 2.04)	4.95 (−1.42, 11.32)	−0.22 (−0.46, 0.02)
LPC 16:1e	−1.05 (−2.07, −0.02)	−0.07 (−0.20, 0.06)	1.83 (−1.89, 5.54)	1.59 (−1.67, 4.86)	0.37 (−0.87, 1.61)	−2.77 (−8.09, 2.56)	−0.24 (−0.44, −0.04)
LPC 18:1	−1.33 (−2.41, −0.25)	0.01 (−0.12, 0.15)	3.43 (−0.49, 7.35)	2.31 (−1.14, 5.77)	0.61 (−0.70, 1.93)	−1.16 (−6.87, 4.55)	−0.24 (−0.45, −0.03)
LPC 18:2	−0.51 (−1.52, 0.50)	0.08 (−0.04, 0.21)	2.56 (−1.05, 6.17)	1.49 (−1.69, 4.67)	0.89 (−0.31, 2.09)	−0.64 (−5.80, 4.52)	−0.26 (−0.45, −0.07)
LPC 20:0	0.09 (−1.17, 1.19)	0.01 (−0.14, 0.16)	6.18 (2.05, 10.30)	4.84 (1.20, 8.49)	1.36 (−0.03, 2.77)	0.11 (−5.89, 6.12)	−0.33 (−0.55, −0.11)
LPC 20:1	−1.01 (−2.03, 0.09)	0.02 (−0.11, 0.15)	3.37 (−0.33, 7.07)	3.37 (0.14, 6.60)	−0.41 (−1.65, 0.83)	−0.36 (−5.72, 5.00)	−0.08 (−0.28, 0.12)
LPC 20:4	−0.52 (−1.52, 0.47)	0.09 (−0.03, 0.22)	2.30 (−1.28, 5.89)	1.72 (−1.43, 4.88)	0.19 (−1.00, 1.38)	0.98 (−4.13, 6.08)	−0.08 (−0.27, 0.11)
LPC 22:6	−0.44 (−1.35, 0.47)	0.07 (−0.04, 0.18)	2.47 (−0.80, 5.75)	2.07 (−0.82, 4.96)	0.30 (−0.79, 1.40)	−0.09 (−4.78, 4.59)	−0.02 (−0.20, 0.15)
PC 34:2e	−0.49 (−1.63, 0.64)	−0.25 (−0.39, −0.11) *	5.65 (1.66, 9.65)	4.42 (0.88, 7.96)	3.21 (1.93, 4.49) **	−10.36 (−15.95, −4.77) ***	−0.37 (−0.58, −0.16)
PC 42:5e	0.22 (−0.80, 1.25)	0.06 (−0.06, 0.19)	2.20 (−1.48, 5.87)	2.41 (−0.81, 5.64)	0.52 (−0.71, 1.75)	−3.41 (−8.62, 1.80)	0.005 (−0.19, 0.20)
SM 42:3	−0.53 (−1.64, 0.58)	−0.02 (−0.16, 0.11)	6.89 (3.05, 10.74)	6.76 (3.47, 10.06) **	0.50 (−0.81, 1.81)	−1.69 (−7.31, 3.94)	0.01 (−0.20, 0.22)
Glycine	0.13 (−0.87, 1.14)	−0.08 (−0.20, 0.05)	−2.91 (−6.51, 0.69)	−1.67 (−4.83, 1.48)	0.41 (−0.78, 1.60)	−5.11 (−10.23, 0.01)	−0.22 (−0.41, −0.03)
Citric acid	−0.80 (−1.93, 0.32)	−0.10 (−0.25, 0.04)	−0.31 (−4.50, 3.87)	0.73 (−2.95, 4.40)	−0.20 (−1.62, 1.22)	−4.81 (−10.73, 1.11)	−0.17 (−0.40, 0.05)

Values are presented as beta estimates (95% confidence interval), and each regression was adjusted for age, sex, body weight change, sagittal diameter change, value for the respective outcome traits at the baseline examination, and the respective metabolite at baseline, and changes in energy intake and physical activity. * Significant after Bonferroni correction for 59 tests. ** Significant after Bonferroni correction for 57 tests. *** Significant after Bonferroni correction for 58 tests. Abbreviations: FAC, fatty acyl chain; LPC, lysophosphatidylcholine; MUFA, monounsaturated fatty acid; PC, phosphatidylcholine; PE, phosphatidylethanolamine; SD, Standard Deviation; SM, sphingomyelin; TG, triglyceride.

**Table 3:** Changes in cardiovascular parameters after the 12-week weight loss maintenance period per 1SD log-transformed changes in the concentrations of metabolites.

Change in Metabolite between 8 Weeks and 12 Weeks	Change in TChol (mg/dL)	Change in LDL-C (mg/dL)	Change in Triglycerides (mg/dL)
TG	3.01 (−0.80, 6.81)	1.54 (−1.59, 4.68)	NA
PC	11.44 (8.13, 14.75) *	8.92 (6.10, 11.74) *	NA
LPC	11.45 (8.17, 14.74) *	8.58 (5.74, 11.41) *	5.66 (0.47, 10.86)
SM	8.30 (4.70, 11.91) *	8.31 (5.49, 11.13) *	NA
FAC	9.82 (6.47, 13.18) *	7.45 (4.65, 10.25) *	10.57 (5.77, 15.36) *
MUFA	6.40 (2.82, 9.97) *	4.86 (1.91, 7.80) *	NA
PC 32:1	NA	NA	16.00 (10.85, 21.14) *
PC 33:1	6.95 (3.34, 10.56) *	NA	15.30 (10.79, 19.82) *
PC 36:1	11.28 (7.83, 14.73) *	NA	8.49 (3.32, 13.67) *
PC 36:4e	8.85 (5.26, 12.44) *	7.12 (4.21, 10.02) *	NA
PC 38:3	13.76 (10.42, 17.09) *	9.84 (6.91, 12.77) *	7.38 (1.93, 12.83)
PC 38:4e	NA	6.55 (3.56, 9.53) *	NA
SM 32:1	12.89 (9.37, 16.37) *	10.08 (7.11, 13.04) *	NA
SM 32:2	11.55 (7.77, 15.34) *	8.12 (4.90, 11.34) *	NA
SM 33:1	10.36 (6.86, 13.86) *	8.94 (6.11, 11.76) *	NA
SM 38:1	8.64 (4.98, 12.31) *	6.71 (3.69, 9.74) *	NA
SM 40:1	11.07 (7.81, 14.32) *	9.71 (7.06, 12.35) *	NA
SM 40:2	11.95 (8.53, 15.37) *	9.57 (6.68, 12.47) *	NA
SM 41:1	12.46 (9.16, 15.76) *	11.17 (8.55, 13.79) *	NA
SM 41:2	8.43 (4.81, 12.05) *	NA	NA
SM 42:1	9.88 (6.48, 13.28) *	8.72 (6.00, 11.45) *	NA
SM 42:3	NA	7.07 (3.92, 10.22) *	NA

Values are presented as beta estimates (95% confidence interval), and each regression was adjusted for age, sex, body weight change, sagittal diameter change, change in the respective outcome traits over 8 weeks, intervention group (satiety controlling foods and control foods), and change in the respective metabolite over 8 weeks. * Significant after Bonferroni correction for 18, 17, and 6 tests for the outcomes TChol, LDL-C, and TG, respectively. NA indicates no analysis performed for the metabolite and adiposity measure because, in Table 2, these parameters were not significantly associated. Abbreviations: FAC, fatty acyl chain; LPC, lysophosphatidylcholine; MUFA, monounsaturated fatty acid; PC, phosphatidylcholine; PE, phosphatidylethanolamine; SD, standard deviation; SM, sphingomyelin; TG, triglyceride.

## Data Availability

Further data will be provided under request to the authors.

## Supplementary Materials

The following are available online at https://www.mdpi.com/article/10.3390/nu13124289/s1. Supplemental Methods: Multiplatform targeted metabolomics, Figure S1: Flow chart of study participants, Table S1: List of metabolites identified, Table S2: Baseline characteristics of 236 participants initially recruited in the SATIN study and 162 participants included in the present analyses, Table S3: Significant changes in concentrations of metabolites after ≥8% weight loss, Table S4: Changes in cardiometabolic parameters at 8 weeks of a low-calorie diet (LCD) per 1SD log-transformed changes in the concentrations of metabolites in a sensitivity analysis with adjustment for changes in energy intake and physical activity, Table S5: Significant changes in concentrations of metabolites after weight loss maintenance.
