# Supplementary material for: Changes in Circulating Metabolites during Weight Loss and Weight Loss Maintenance in Relation to Cardiometabolic Risk
Source: Nutrients. 2021 Nov 27;13(12):4289. doi: 10.3390/nu13124289 (PMC8708084; doi:10.3390/nu13124289)
Supplement: Supplementary file 1 [file nutrients-13-04289-s001.zip › nutrients-1482836-supplementary.pdf]

Supplemental Methods

Multiplatform targeted metabolomics

Automated multiple plasma sample extraction

The Bravo Automated Liquid Handling Platform from Agilent Technologies was used to extract plasma samples in 96-well format plates. For GC-HRMS analysis, a protein precipitation extraction will be made by adding 400 of µlMeOH: H2O (8: 1) mixture to a volume of 100 µl of plasma. The mixture was stirred and centrifuged and the supernatants were collected in new 96 well plates that contain internal standard mixture. This plate was evaporated to dryness with a vacuum centrifugation system (Speed Vac) and dried extracts were reconstituted with 30 µL of methoxyamine and incubated during 90 min at 37ºC. Finally, the metabolites were sylilated with 45 µL of MSTFA + 1 % TMCS at room temperature during 60 min.

For LC-HRMS analysis and NMR analyses, lipidic fraction was obtained by a liquid-liquid extraction using a methanol/methyl-tert-butyl ether mixture. These solvents were automatically and sequentially added to a volume of 100 µl of plasma with agitation stages between them and final centrifugation to promote phase separation. Then, a small aliquot of the supernatant (organic phase) was dispensed and diluted 1:10 with methanol in a new 96-well plates containing deuterated internal standards for each family of lipids (Lipidomix SPLASH from Avanti Polar Lipid) for lipidomic analysis using liquid chromatography coupled to a time of flight high resolution mass spectrometry (LC-HRMS).

For lipidomic analysis by Nuclear Magnetic Resonance (NMR), a second aliquot of the supernatant (organic phase) was dispensed in new 96 well plates that was evaporated to dryness with Speed Vac. Afterwards, they were reconstituted with a solution of

CD3Cl: CD3OD with 4% D2O and 0.01% TMS (0.067 mM, Eretic Signal 6,166 mM) and analyzed by proton NMR (^{1}H-NMR).

GC-HRMS metabolomics profiling

Samples were analyzed in a 7890A Series gas chromatograph coupled to a 7000 GC-qTOF from Agilent Technologies using a chromatographic column J&W Scientific HP5-MS (30 m x 0.25 mm i.d., 0.25 µm film) (Agilent Technologies), and helium as a carrier gas. Ionization was done by electronic impact (EI), recording data in Full Scan mode. Quantification was performed by internal standard calibration, using the corresponding analytical standard for each determined metabolite and a deuterated internal standard depending on the family of metabolite. Internal standards used were succinic acid-d4, glycerol-13C3, norvaline, L-methionine-(carboxy-13C, methyl-d3), D-glucose-13C6, myristic-d27 acid and alpha-tocopherol-d6.

LC-HRMS lipidomics profiling

The lipid species were determined using UHPLC-qTOF from Agilent Technologies (6550). The ionization was performed in positive electrospray and mass calibration reference was used along all the analyses to maintain the mass accuracy below 5 ppm. Lipids were separated on C18 reversed phase column (Kinetex C18-EVO, Phenomenex) and a ternary mobile phase (water/methanol/2-propanol) was used. The quantification of each lipid was made by an internal standard calibration method using one analytical standard and one deuterated internal standard for each lipid family (lysophosphatidylcholines, phosphatidylcholines, sphingomyelins, and triglycerides).

^{1}H-NMR lipidomic profiling

^{1}H NMR spectra were recorded at 300K on an Avance III 600 spectrometer (Bruker®, Germany) operating at a proton frequency of 600.20 MHz using a 5 mm PBBO gradient probe. Lipidic samples were measured and recorded in procno 11 using a simple

presaturation sequence (RD–90 °–ACQ zgpr pulse program in Bruker®) to eliminate the residual water moisture of deuterated methanol. Solvent presaturation with irradiation power of 50 Hz was applied during recycling delay (RD = 5 s) and mixing time. The 90 ° pulse length was calibrated for each sample and varied from 10.42 to 11.49 ms. The spectral width was 12 kHz (20 ppm), and a total of 64 transients were collected into 64 k data points for each ^{1}H spectrum. The exponential line broadening applied before Fourier transformation was of 0.3 Hz. The frequency domain spectra were manually phased and baseline-corrected using TopSpin software (version 2.1, Bruker). After pre-processing and visual checking of NMR dataset, specific ^{1}H regions of diacylglycerols, triglycerides and total lipids based on terminal methyl and methylene signals were identified by in the spectra using a comparison into AMIX 3.9 software. Curated identified regions across the spectra were integrated using the same AMIX 3.9 software package and exported to Excel spreadsheet in order to give absolute concentrations.

Metabolite quantification and data normalization in MS analysis: A pooled matrix sample generated by taking a small volume of each experimental sample served as a technical replicate throughout the data set. As study spanning multiple days, a data normalization step is performed to correct variation resulting from instrument inter-day tuning differences. Essentially, each compound was corrected in run-day blocks through quality controls (QCs) normalizing each data point proportionately.

Metabolite quantification and data normalization in NMR analysis: The calibration of the quantification in NMR was performed using the PULCON (PULse length-based CONcentration determination) methodology (ERETIC®), which is based on the principle of reciprocity. The stability, reproducibility and quantitative nature of the NMR technique allows the use of PULCON method to quantify different batches of

samples without any further quality control correction. The PULCON method requires only one measurement of an external reference sample (in eg. 2mM sucrose solution in D2O + H2O) used to calibrate ERETIC signal for all samples. This reference virtual signal, thanks to the stability of modern NMR instruments, can be used for several months.

Figure S1 Flow chart of study participants

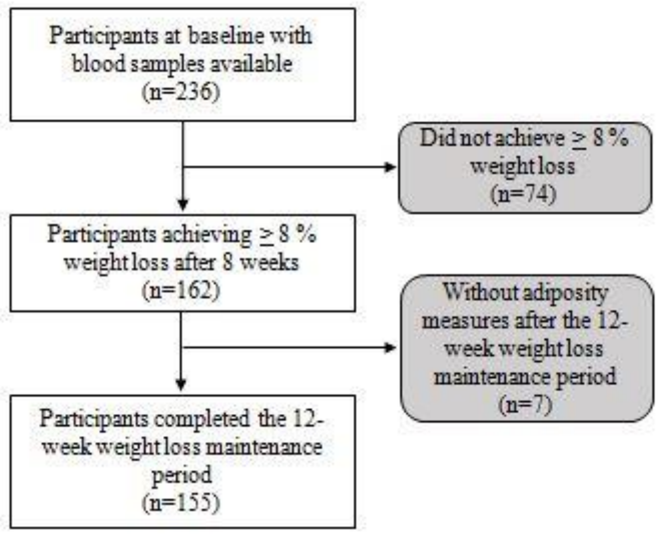

Table S1 List of metabolites identified

Metabolite	Platform	Quantitative ion (m/z)	Retention Time (min)	Repeatability (% RSD, n=5)*	Reproducibility (% RSD, n=3)*
2-Hydroxybutanoic acid in plasma	GC-MS	131.0895	6.9	3.4	4.2
3-Hydroxybutanoic acid in plasma	GC-MS	117.0731	7.3	2.0	2.7
Alanine in plasma	GC-MS	116.0916	6.5	3.6	3.7
alpha-Tocopherol in plasma	GC-MS	237.1337	26.3	2.5	7.6
Cholesterol in plasma	GC-MS	129.0746	26.4	4.8	11.9
Citric acid in plasma	GC-MS	273.105	15.7	1.4	8.0
Fructose in plasma	GC-MS	307.165	16.4	3.7	19.7
Gliceric acid in plasma	GC-MS	189.0782	9.8	3.3	8.0
Glucose in plasma	GC-MS	319.1592	16.6	4.6	19.8
Glutamic acid in plasma	GC-MS	246.1392	13.4	2.2	4.2
Glycerol in plasma	GC-MS	218.1185	9	5.5	6.1
Glycine in plasma	GC-MS	174.1153	9.4	1.4	1.9
Glycolic acid in plasma	GC-MS	177.0764	6	2.2	11.8
Isoleucine in plasma	GC-MS	158.1404	9.2	1.5	3.4
Lactic acid in plasma	GC-MS	190.088	5.8	3.6	5.6
Leucine in plasma	GC-MS	158.1432	8.9	1.7	1.7
Linoleic acid in plasma	GC-MS	117.0382	19.2	6.5	16.5
Lysine in plasma	GC-MS	200.1102	16	8.5	18.7
Methionine in plasma	GC-MS	128.0909	12.2	4.4	3.9
Oleic acid in plasma	GC-MS	117.0382	19.3	2.4	13.6
Ornithine in plasma	GC-MS	142.1063	15.6	3.3	9.4
Palmitic acid in plasma	GC-MS	117.0382	17.7	4.7	9.3
Phenylalanine in plasma	GC-MS	218.105	13.5	2.8	0.5
Proline in plasma	GC-MS	142.1082	9.3	6.4	22.5
Serine in plasma	GC-MS	204.1316	10.2	1.4	2.0
Stearic acid in plasma	GC-MS	117.0382	19.5	2.9	17.6
Threonine in plasma	GC-MS	218.1053	10.6	1.2	2.8
Tryptophan in plasma	GC-MS	202.1134	19.5	7.9	14.3
Tyrosine in plasma	GC-MS	179.0902	16.9	3.8	26.2
Valine in plasma	GC-MS	144.1229	8.1	2.3	2.7
Lysophosphatidylcholine 14:0 in plasma	LC-MS	468.3085	1.3	3.8	0.4
Lysophosphatidylcholine 15:0 in plasma	LC-MS	482.3241	1.5	3.7	0.8

Lysophosphatidylcholine 16:0 e in plasma	LC-MS	482.3605	1.9	4.3	2.1
Lysophosphatidylcholine 16:0 in plasma	LC-MS	496.3398	1.8	4.2	1.0
Lysophosphatidylcholine 16:1 e in plasma	LC-MS	480.3449	2	3.1	6.2
Lysophosphatidylcholine 16:1 in plasma	LC-MS	494.3241	1.5	5.6	7.5
Lysophosphatidylcholine 17:0 in plasma	LC-MS	510.3554	2	4.3	1.2
Lysophosphatidylcholine 18:0 e in plasma	LC-MS	510.3918	2.5	3.8	1.2
Lysophosphatidylcholine 18:0 in plasma	LC-MS	524.3711	2.2	2.3	1.5
Lysophosphatidylcholine 18:1 in plasma	LC-MS	522.3554	1.9	3.1	1.2
Lysophosphatidylcholine 18:2 in plasma	LC-MS	520.3398	1.6	3.0	1.1
Lysophosphatidylcholine 20:0 in plasma	LC-MS	552.4024	2.7	4.1	1.5
Lysophosphatidylcholine 20:1 in plasma	LC-MS	550.3867	2.3	3.8	1.6
Lysophosphatidylcholine 20:3 in plasma	LC-MS	546.3554	1.8	5.1	2.3
Lysophosphatidylcholine 20:4 in plasma	LC-MS	544.3398	1.7	4.9	1.8
Lysophosphatidylcholine 22:6 in plasma	LC-MS	568.3398	1.7	3.9	1.2
Phosphatidylcholine 30:0 in plasma	LC-MS	706.5381	4.1	2.9	2.3
Phosphatidylcholine 32:0 in plasma	LC-MS	734.5694	4.7	4.3	3.1
Phosphatidylcholine 32:1 e in plasma	LC-MS	718.5745	4.7	4.2	3.2
Phosphatidylcholine 32:1 in plasma	LC-MS	732.5538	4.2	3.9	2.7
Phosphatidylcholine 32:2 in plasma	LC-MS	730.5381	3.9	3.2	2.5
Phosphatidylcholine 33:1 in plasma	LC-MS	746.5694	4.5	3.7	3.0
Phosphatidylcholine 34:0 in plasma	LC-MS	762.6008	5.5	3.7	3.6
Phosphatidylcholine 34:1 e in plasma	LC-MS	746.6058	5.4	7.5	3.2
Phosphatidylcholine 34:2 e in plasma	LC-MS	744.5918	5.1	3.6	2.8
Phosphatidylcholine 34:2 in plasma	LC-MS	758.5694	4.5	3.6	2.8
Phosphatidylcholine 34:3 e in plasma	LC-MS	742.5778	4.8	3.3	2.7
Phosphatidylcholine 34:4 in plasma	LC-MS	754.5381	4	4.2	2.4
Phosphatidylcholine 35:1 in plasma	LC-MS	774.6007	5.3	3.8	3.5
Phosphatidylcholine 35:2 in plasma	LC-MS	772.5851	5	2.6	3.2
Phosphatidylcholine 36:1 in plasma	LC-MS	788.6164	5.8	7.8	3.8
Phosphatidylcholine 36:2 e in plasma	LC-MS	772.6215	5.1	2.7	3.6
Phosphatidylcholine 36:3 in plasma	LC-MS	784.5851	4.8	3.2	3.0
Phosphatidylcholine 36:4 e in plasma	LC-MS	768.5902	5	3.2	3.0
Phosphatidylcholine 36:5 e in plasma	LC-MS	766.5762	4.7	3.5	2.8
Phosphatidylcholine 36:5 in plasma	LC-MS	780.5538	4.3	3.5	2.8
Phosphatidylcholine 37:4 in plasma	LC-MS	796.5851	5	4.5	2.8
Phosphatidylcholine 38:3 in plasma	LC-MS	812.6164	5.6	7.3	3.7
Phosphatidylcholine 38:4 e in plasma	LC-MS	796.6215	5.9	2.6	3.6
Phosphatidylcholine 38:4 in plasma	LC-MS	810.6007	5.4	2.6	3.6

Phosphatidylcholine 38:5 e in plasma	LC-MS	794.6058	5.2	3.6	3.4
Phosphatidylcholine 38:5 in plasma	LC-MS	808.5851	5	3.6	3.4
Phosphatidylcholine 38:6 in plasma	LC-MS	806.5694	4.4	5.0	3.0
Phosphatidylcholine 40:4 e in plasma	LC-MS	824.6528	6.4	3.9	4.1
Phosphatidylcholine 40:4 in plasma	LC-MS	838.632	6	3.9	4.1
Phosphatidylcholine 40:5 e in plasma	LC-MS	822.6371	5.9	4.9	3.8
Phosphatidylcholine 40:6 in plasma	LC-MS	834.6007	5.4	3.8	3.6
Phosphatidylcholine 42:5 e in plasma	LC-MS	850.6684	6.4	5.3	4.9
Phosphatidylethanolamine 36:5 e in plasma	LC-MS	724.5276	4.4	7.6	4.9
Phosphatidylethanolamine 38:5 e in plasma	LC-MS	752.5589	5.5	7.7	6.1
Phosphatidylethanolamine 38:6 e in plasma	LC-MS	750.5449	5.2	6.4	4.7
Sphingomyelin 32:1 in plasma	LC-MS	675.5436	3.5	4.6	1.8
Sphingomyelin 32:2 in plasma	LC-MS	673.5279	3.3	3.1	1.7
Sphingomyelin 33:1 in plasma	LC-MS	689.5592	3.7	3.0	2.1
Sphingomyelin 34:1 in plasma	LC-MS	703.5749	4	5.3	2.2
Sphingomyelin 34:2 in plasma	LC-MS	701.5592	3.7	2.6	2.1
Sphingomyelin 35:1 in plasma	LC-MS	717.5905	4.4	3.0	2.6
Sphingomyelin 36:0 in plasma	LC-MS	733.6218	4.8	11.6	3.0
Sphingomyelin 36:1 in plasma	LC-MS	731.6062	4.6	3.6	2.6
Sphingomyelin 36:2 in plasma	LC-MS	729.5905	4.2	4.8	3.1
Sphingomyelin 38:1 in plasma	LC-MS	759.6375	5.4	3.1	3.5
Sphingomyelin 38:2 in plasma	LC-MS	757.6218	4.8	3.1	3.0
Sphingomyelin 40:1 in plasma	LC-MS	787.6688	6.2	4.4	4.2
Sphingomyelin 40:2 in plasma	LC-MS	785.6531	5.7	4.3	3.8
Sphingomyelin 41:1 in plasma	LC-MS	801.6844	6.3	4.1	3.2
Sphingomyelin 41:2 in plasma	LC-MS	799.6688	6.1	3.8	4.1
Sphingomyelin 42:1 in plasma	LC-MS	815.7001	6.5	2.1	1.3
Sphingomyelin 42:2 in plasma	LC-MS	813.6844	6.2	4.2	4.8
Sphingomyelin 42:3 in plasma	LC-MS	811.6688	5.8	4.2	5.6
Triacylglycerides 50:1 in plasma	LC-MS	850.7858	9.7	13.6	5.7
Triacylglycerides 50:2 in plasma	LC-MS	848.7702	9.3	12.2	5.4
Triacylglycerides 50:3 in plasma	LC-MS	846.7545	8.9	6.2	5.3
Triacylglycerides 52:2 in plasma	LC-MS	876.8015	9.8	16.9	7.7
Triacylglycerides 52:3 in plasma	LC-MS	874.7858	9.4	8.4	6.3
Triacylglycerides 52:4 in plasma	LC-MS	872.7702	9.1	15.8	5.7
Triacylglycerides 54:2 in plasma	LC-MS	904.8328	10.3	12.6	8.2
Triacylglycerides 54:3 in plasma	LC-MS	902.8171	10	13.7	7.9
Triacylglycerides 54:4 in plasma	LC-MS	900.8015	9.6	15.1	6.7

Triacylglycerides 54:5 in plasma	LC-MS	898.7858	9.2	5.3	5.8
Fatty acyl chains in serum	NMR	-	-	3.3	9.1
Total Cholesterol in serum	NMR	-	-	3.8	10.4
Esterified Cholesterol in serum	NMR	-	-	4.0	10.1
Phosphatidyl Choline in serum	NMR	-	-	2.9	9.6
Lysophosphatidyl Choline in serum	NMR	-	-	2.7	7.9
Free Cholesterol in serum	NMR	-	-	3.5	11.2
Triglycerides in serum	NMR	-	-	3.6	10.2
Sphingomyelin in serum	NMR	-	-	2.9	12.6
monounsaturated fatty acids in serum	NMR	-	-	3.9	11.3
polyunsaturated fatty acids in serum	NMR	-	-	3.7	11.5
Linoleic in serum	NMR	-	-	4.0	11.3
Arachidonic acid + Eicosapentaenoic acid in serum	NMR	-	-	3.8	4.4
Omega-3 in serum	NMR	-	-	3.8	4.4
Docosahexaenoic acid in serum	NMR	-	-	3.7	6.8

*Repeatability and reproducibility (expressed as RSD) were evaluated with a pool of well-characterized human plasma prepared and analyzed on the same day (repeatability) and three different days (reproducibility).

Table S2. Baseline characteristics of 236 participants initially recruited in the SATIN study and 162 participants included in the present analyses

Variables	n=236	n=162
**Sex** (%Women)	78.8	75.0
**Age** (years)	46.4	47.5 ± 9.9
**Weight** (kg)	87.5 ± 11.2	88.1 ± 10.7
**BMI** (kg/m^2)	31.1 ± 2.2	30.9 ± 2.0
**Sagittal diameter** (cm)	23.1 ± 2.3	23.2 ± 2.4
**Glucose** (mg/dL)	93.3 ± 11.0	94.9 ± 11.2
**Insulin** (mcUI/mL)	10.2 ± 8.8	9.1 ± 6.4
**HOMA-IR**	2.4 ± 2.2	2.2 ± 1.7
**TChol** (mg/dL)	196.0 ± 34.9	197.6 ± 34.9
**HDL-C** (mg/dL)	55.7 ± 15.3	56.3 ± 15.9
**LDL-C** (mg/dL)	119.9 ± 30.5	120.6 ± 30.9
**Triglycerides** (mg/dL)	102.3 ± 48.9	103.1 ± 50.5
**IL-6** (pg/mL)	2.54 ± 3.95	2.24 ± 3.43
**CRP** (mg/mL)	1.43 ± 1.52	1.41 ± 1.61
**EI** (Kcal/d)	1922.2 ± 623.7	1953.5 ± 633.5
**TPA** (CPM)	622.2 ± 192.5	608.9 ± 187.9

Abbreviations: BMI, body mass index; CPM, counts/min; CRP, C-reactive protein; EI, energy intake; HDL-C, high-density lipoprotein-cholesterol; IL-6, interleukin 6; LCD, low-calorie diet; LDL-C, low-density lipoprotein-cholesterol; TChol, total cholesterol; TPA, total physical activity.

Table S3 Significant changes in concentrations of metabolites after ≥8% weight loss

Change in metabolite	Metabolite	Mean (95%CI)	Units
Decreased Concentrations	Free Cholesterol	0.042 (0.03, 0.05)	mM
	Esterified Cholesterol	0.054 (0.04, 0.06)	mM
	Total Cholesterol	0.050 (0.04, 0.06)	mM
	Triglycerides	0.047 (0.03, 0.06)	mM
	Phosphatidylcholine	0.042 (0.03, 0.05)	mM
	Lysophosphatidylcholine	0.044 (0.03, 0.05)	mM
	Sphingomyelin	0.020 (0.009, 0.03)	mM
	Fatty acyl chains	0.040 (0.03, 0.05)	mM
	Monounsaturated fatty acids	0.006 (0.003, 0.01)	% of fatty acid chains
	LPC 14:0	0.003 (0.002, 0.004)	µg/dL
	LPC 20:3	0.002 (0.001, 0.004)	µg/dL
	PC 30:0	0.025 (0.02, 0.03)	µg/dL
	PC 32:1	0.072 (0.05, 0.09)	µg/dL
	PC 32:2	0.024 (0.02, 0.03)	µg/dL
	PC 33:1	0.014 (0.01, 0.02)	µg/dL
	PC 34:4	0.013 (0.01, 0.02)	µg/dL
	PC 35:1	0.009 (0.006, 0.01)	µg/dL
	PC 36:1	0.060 (0.04, 0.08)	µg/dL
	PC 36:4e	0.027 (0.02, 0.04)	µg/dL
	PC 36:5	0.049 (0.02, 0.07)	µg/dL
	PC 38:3	0.117 (0.09, 0.13)	µg/dL
	PC 38:4	0.031 (0.02, 0.04)	µg/dL
	PC 38:4e	0.015 (0.01, 0.02)	µg/dL
	PC 40:4	0.008 (0.005, 0.01)	µg/dL
	PC 40:6	0.033 (0.02, 0.04)	µg/dL
	PE 36:5e	0.042 (0.03, 0.05)	µg/dL
	PE 38:5e	0.046 (0.04, 0.05)	µg/dL
	PE 38:6e	0.039 (0.03, 0.05)	µg/dL
	SM 32:1	0.034 (0.03, 0.04)	µg/dL
	SM 32:2	0.003 (0.003, 0.004)	µg/dL
	SM 33:1	0.006 (0.004, 0.008)	µg/dL
	SM 35:1	0.002 (0.001, 0.003)	µg/dL
	SM 36:0	0.003 (0.001, 0.004)	µg/dL
	SM 36:1	0.012 (0.006, 0.02)	µg/dL
	SM 38:1	0.035 (0.003)	µg/dL
	SM 40:1	0.040 (0.03, 0.04)	µg/dL
	SM 40:2	0.016 (0.01, 0.02)	µg/dL
	SM 41:1	0.043 (0.04, 0.05)	µg/dL
	SM 41:2	0.011 (0.009, 0.01)	µg/dL
	SM 42:1	0.032 (0.02, 0.04)	µg/dL
	TG 50:2	0.144 (0.007, 0.22)	µg/dL
	Lactic acid	0.055 (0.03, 0.08)	mM
	Glycolic acid	0.022 (0.01, 0.03)	mM
	Valine	0.027 (0.01, 0.04)	mM
	Glutamate	0.074 (0.05, 0.10)	mM
	Glucose	0.012 (0.006, 0.02)	mM
	Tyrosine	0.033 (0.02, 0.05)	mM
Increased Concentrations	LPC 16:0	0.027 (0.04, 0.04)	µg/dL
	LPC 16:1e	0.002 (0.001, 0.004)	µg/dL
	LPC 18:1	0.023 (0.01, 0.03)	µg/dL
	LPC 18:2	0.030 (0.02, 0.04)	µg/dL
	LPC 20:0	0.0002 (0.0001, 0.0003)	µg/dL
	LPC 20:1	0.0008 (0.0005, 0.0009)	µg/dL
	LPC 20:4	0.006 (0.004, 0.008)	µg/dL
	LPC 22:6	0.002 (0.001, 0.003)	µg/dL
	PC 34:2e	0.004 (0.002, 0.006)	µg/dL
	PC 42:5e	0.002 (0.001, 0.003)	µg/dL
	SM 42:3	0.016 (0.01, 0.02)	µg/dL
	Glycine	0.068 (0.05, 0.08)	mM
	Citric acid	0.053 (0.03, 0.07)	mM

Significance was calculated using paired t-test with Bonferroni correction for 123 tests. Abbreviations: CI, confidence interval; LPC, lysophosphatidylcholine; PC, phosphatidylcholine; PE, phosphatidylethanolamine; SM, sphingomyelin; TG, triglyceride.

Table S4 Changes in cardiometabolic parameters at 8 weeks of a low-calorie diet (LCD) per 1SD log-transformed changes in the concentrations of metabolites in a sensitivity analysis with adjustment for changes in energy intake and physical activity

**Change in metabolite between baseline and 8 weeks LCD**	**Change in TChol (mg/dL)**	**Change in LDL-C (mg/dL)**	**Change in HDL-C (mg/dL)**	**Change in Triglycerides (mg/dL)**
Free Chol	NA	NA	NA	6.85 (1.39, 12.31)
Esterified Chol	NA	NA	NA	5.51 (0.37, 10.64)
Total Chol	NA	NA	NA	-1.60 (-6.83, 3.64)
TG	10.66 (6.84, 14.48)*	6.82 (3.24, 10.39)*	-0.87 (-2.25, 0.51)	NA
PC	13.19 (9.78, 16.59)*	9.61 (6.39, 12.82)*	2.05 (0.72, 3.38)	7.32 (1.88, 12.75)
LPC	13.29 (9.94, 16.63)*	8.93 (5.69, 12.18)*	2.52 (1.22, 3.82)*	8.61 (3.25, 13.97)
SM	8.88 (4.78, 12.99)*	8.12 (4.56, 11.68)*	0.24 (-1.19, 1.67)	2.56 (-3.21, 8.33)
FAC	14.05 (10.29, 17.80)*	10.03 (6.49, 13.57)*	0.81 (-0.68, 2.29)	15.60 (10.17, 21.03)**
MUFA	8.48 (5.28, 11.67)*	7.75 (4.93, 10.57)*	0.52 (-0.68, 1.72)	3.93 (-0.91, 8.78)
LPC 14:0	5.44 (0.22, 10.65)	2.28 (-2.39, 6.94)	1.24 (-0.55, 3.03)	6.44 (0.78, 13.66)
LPC 20:3	6.35 (2.01, 10.70)	4.20 (0.24, 8.15)	1.19 (-0.35, 2.73)	4.48 (-1.76, 10.71)
PC 30:0	7.01 (1.76, 12.27)	3.20 (-1.57, 7.98)	0.91 (-0.94, 2.77)	9.93 (2.52, 17.35)
PC 32:1	5.89 (1.65, 10.12)	1.61 (-2.27, 5.49)	1.18 (-0.32, 2.68)	12.09 (6.19, 17.98)**
PC 32:2	7.07 (2.38, 11.75)	2.89 (-1.38, 7.16)	2.08 (0.48, 3.69)	9.38 (2.99, 15.77)
PC 33:1	8.32 (3.73, 12.90)*	4.80 (0.60, 9.00)	0.25 (-1.41, 1.92)	13.75 (7.52, 19.99)**
PC 34:4	7.69 (2.70, 12.68)	3.29 (-1.25, 7.82)	2.40 (0.70, 4.11)	9.51 (2.65, 16.38)
PC 35:1	10.80 (6.52, 15.09)*	8.36 (4.50, 12.23)*	-0.20 (-1.76, 1.36)	10.97 (4.93, 17.02)**
PC 36:1	9.06 (4.51, 13.61)*	5.67 (1.47, 9.86)	1.05 (-0.60, 2.70)	9.56 (3.01, 16.12)
PC 36:4e	9.51 (4.76, 14.27)*	8.15 (3.95, 12.36)*	2.56 (0.90, 4.21)	-6.56 (-13.31, 0.19)
PC 36:5	3.93 (0.22, 7.64)	2.39 (-0.95, 5.72)	0.79 (-0.50, 2.08)	2.24 (-2.95, 7.43)
PC 38:3	10.08 (6.25, 13.90)*	6.68 (3.09, 10.27)*	0.92 (-0.51, 2.36)	12.78 (7.35, 18.21)*
PC 38:4	6.82 (2.71, 10.93)	5.55 (1.65, 9.46)	0.25 (-1.20, 1.70)	7.07 (1.07, 13.08)
PC 38:4e	8.23 (4.03, 12.42)*	7.24 (3.52, 10.97)*	2.13 (0.66, 3.60)	-6.65 (-12.64, -0.66)
PC 40:4	7.08 (2.56, 11.59)	3.68 (-0.45, 7.81)	1.82 (0.24, 3.39)	8.32 (2.04, 14.60)
PC 40:6	8.10 (4.48, 11.72)*	5.00 (1.67, 8.33)	0.70 (-0.62, 2.02)	10.03 (4.99, 15.07)**
PE 36:5e	1.59 (-2.98, 6.17)	1.36 (-2.70, 5.42)	1.13 (-0.42, 2.67)	-3.79 (-10.06, 2.48)
PE 38:5e	3.22 (-1.17, 6.93)	3.05 (-0.90, 6.99)	1.35 (-0.15, 2.85)	-6.22 (-12.27, -0.16)
PE 38:6e	4.00 (-0.90, 8.90)	3.05 (-1.31, 7.41)	2.60 (0.98, 4.22)	-7.06 (-13.76, -0.36)
SM 32:1	14.16 (10.28, 18.04)*	11.79 (8.29, 15.29)*	2.13 (0.63, 3.63)	1.49 (-4.74, 7.71)
SM 32:2	11.20 (6.64, 15.76)*	8.05 (3.86, 12.25)*	1.86 (0.24, 3.48)	8.76 (2.09, 15.42)
SM 33:1	12.34 (8.52, 16.17)*	10.99 (7.61, 14.37)*	1.62 (0.17, 3.07)	-0.23 (-6.23, 5.76)
SM 35:1	11.06 (7.40, 14.73)*	9.60 (6.36, 12.85)*	1.28 (-0.09, 2.66)	-0.29 (-5.76, 5.17)
SM 36:0	6.99 (1.73, 12.24)	7.80 (3.25, 12.35)	-0.60 (-2.40, 1.19)	0.34 (-6.82, 7.50)
SM 36:1	9.99 (5.97, 14.01)*	8.78 (5.27, 12.29)*	1.03 (-0.40, 2.47)	1.77 (-4.11, 7.65)
SM 38:1	10.47 (6.95, 13.99)*	8.06 (4.83, 11.28)*	1.44 (0.12, 2.75)	5.90 (0.57, 11.23)
SM 40:1	14.08 (10.21, 17.95)*	11.36 (7.81, 14.92)*	2.48 (1.02, 3.94)	6.15 (0.02, 12.28)

SM 40:2	14.07 (10.23, 17.90)*	11.04 (7.53, 14.55)*	2.65 (1.21, 4.08)*	3.44 (-2.72, 9.60)
SM 41:1	15.33 (11.39, 19.28)*	12.57 (8.94, 16.20)*	2.20 (0.64, 3.76)	5.88 (-0.55, 12.32)
SM 41:2	9.32 (3.89, 14.75)	7.19 (2.30, 12.07)	1.68 (-0.21, 3.57)	4.74 (-2.94, 12.43)
SM 42:1	11.96 (7.54, 16.37)*	8.87 (4.76, 12.97)*	2.44 (0.87, 4.01)	7.88 (1.41, 14.35)
TG 50:2	6.41 (2.50, 10.32)*	4.02 (0.48, 7.56)	-1.19 (-2.51, 0.12)	NA
Lactic acid	3.49 (-0.64, 7.62)	1.94 (-1.76, 5.64)	0.63 (-0.79, 2.06)	4.75 (-0.94, 10.45)
Glycolic acid	0.35 (-4.19, 4.90)	-0.19 (-4.18, 3.80)	0.15 (-1.39, 1.69)	-2.03 (-8.41, 4.36)
Valine	7.98 (4.13, 11.83)*	6.56 (3.14, 9.99)*	-0.25 (-1.65, 1.14)	7.34 (1.79, 12.90)
Glutamate	-0.54 (-4.43, 3.35)	-0.78 (-4.22, 2.66)	-0.15 (-1.48, 1.18)	2.04 (-3.23, 7.31)
Glucose	1.97 (-2.27, 6.20)	1.65 (-2.11, 5.42)	0.52 (-0.92, 1.96)	-0.81 (-6.69, 5.06)
Tyrosine	5.52 (1.34, 9.70)	3.66 (-0.12, 7.43)	1.41 (-0.04, 2.86)	2.24 (-3.71, 8.19)
LPC 16:0	5.97 (1.14, 10.80)	4.61 (0.29, 8.94)	0.70 (-0.97, 2.37)	1.98 (-4.80, 8.77)
LPC 16:1e	1.24 (-2.76, 5.24)	1.42 (-2.13, 4.97)	0.43 (-0.93, 1.79)	-4.70 (-10.20, 0.79)
LPC 18:1	3.25 (-1.07, 7.56)	2.31 (-1.51, 6.13)	0.90 (-0.56, 2.36)	-4.03 (-10.08, 2.02)
LPC 18:2	2.63 (-1.30, 6.56)	1.71 (-1.78, 5.20)	1.22 (-0.11, 2.54)	-2.97 (-8.40, 2.45)
LPC 20:0	5.03 (0.53, 9.54)	4.10 (0.10, 8.11)	1.70 (0.17, 3.24)	-2.99 (-9.27, 3.28)
LPC 20:1	2.85 (-1.22, 6.91)	3.09 (-0.48, 6.66)	-0.14 (-1.53, 1.24)	-2.62 (-8.25, 3.01)
LPC 20:4	2.35 (-1.42, 6.13)	1.82 (-1.53, 5.17)	0.50 (-0.78, 1.78)	-0.61 (-5.82, 4.61)
LPC 22:6	2.39 (-1.03, 5.80)	2.22 (-0.82, 5.25)	0.42 (-0.74, 1.59)	-1.63 (-6.35, 3.08)
PC 34:2e	5.17 (0.82, 9.51)	3.84 (-0.05, 7.74)	3.31 (1.90, 4.72)*	-10.57 (-16.42, -4.72)**
PC 42:5e	2.14 (-1.75, 6.03)	2.46 (-0.97, 5.90)	0.59 (-0.73, 1.91)	-3.67 (-8.99, 1.66)
SM 42:3	7.39 (3.25, 11.53)	7.47 (3.90, 11.04)*	0.22 (-1.24, 1.67)	-1.29 (-7.20, 4.62)
Glycine	-3.50 (-7.41, 0.41)	-2.72 (-6.18, 0.74)	0.80 (-0.52, 2.12)	-4.72 (-10.14, 0.69)
Citric acid	0.85 (-3.54, 5.25)	1.56 (-2.34, 5.45)	-0.03 (-1.57, 1.50)	-3.73 (-9.83, 2.36)

Values presented as beta estimates (95% confidence interval) and each regression was adjusted for age, sex, body weight change, sagittal diameter change, value for the respective outcome traits at the baseline examination, and the respective metabolite at baseline, and changes in energy intake and physical activity. *Significant after Bonferroni correction for 57 tests. ** Significant after Bonferroni correction for 58 tests. Abbreviations: FAC, fatty acyl chain; LPC, lysophosphatidylcholine; MUFA, monounsaturated fatty acid; PC, phosphatidylcholine; PE, phosphatidylethanolamine; SD, Standard Deviation; SM, sphingomyelin; TG, triglyceride.

Table S5 Significant changes in concentrations of metabolites after weight loss maintenance

	**Metabolite**	**Mean (95%CI)**	**Units**
Increased Concentrations	Triglycerides	4.23 (4.17, 4.28)	mM
	Phosphatidylcholine	0.53 (0.50, 0.55)	mM
	Lysophosphatidylcholine	0.41 (0.38, 0.43)	mM
	Fatty acyl chains	2.65 (2.63, 2.67)	mM
	PC 32:1	0.04 (0.02, 0.06)	mM
	PC 33:1	3.90 (3.89, 3.90)	µg/dL
	PC 36:1	4.99 (4.95, 5.03)	µg/dL
	PC 36:4e	4.49 (4.46, 4.51)	µg/dL
	PC 38:3	4.97 (4.93, 5.01)	µg/dL
	PC 38:4e	4.11 (4.10, 4.12)	µg/dL
	SM 32:1	4.29 (4.28, 4.31)	µg/dL
	SM 32:2	4.01 (4.009, 4.01)	µg/dL
	SM 33:1	4.06 (4.05, 4.06)	µg/dL
	SM 38:1	4.18 (4.16, 4.19)	µg/dL
	SM 40:1	4.23 (4.22, 4.25)	µg/dL
	SM 40:2	4.25 (4.24, 4.26)	µg/dL
	SM 41:1	4.19 (4.18, 4.20)	µg/dL
	SM 41:2	3.95 (3.94, 3.95)	µg/dL
	SM 42:1	4.06 (4.05, 4.07)	µg/dL
	SM 42:3	4.38 (4.37, 4.40)	µg/dL
Decreased Concentrations	Sphingomyelin	0.25 (0.23, 0.28)	mM
	Monounsaturated fatty acids	0.47 (1.45, 1.48)	% of fatty acid chains

Significance was calculated using paired t-test with Bonferroni correction for 28 tests. Abbreviations: CI; confidence interval, LPC, lysophosphatidylcholine; PC, phosphatidylcholine; PE, phosphatidylethanolamine; SM, sphingomyelin.
